# The effects of the COVID-19 pandemic on the diagnosis and prognosis of melanoma 2 years after the pandemic in two Romanian counties

**DOI:** 10.3389/fmed.2024.1328488

**Published:** 2024-01-23

**Authors:** Adina Patricia Apostu, Ștefan Cristian Vesa, Simona Frățilă, Gabriela Iancu, Nona Bejinariu, Maximilian Muntean, Simona C. Șenilă, Oana Alexandra Baba, Cristina Pop Secășan, Loredana Ungureanu

**Affiliations:** ^1^Department of Dermatology, “Iuliu Hațieganu” University of Medicine and Pharmacy, Cluj-Napoca, Romania; ^2^Clinical Hospital of Infectious Diseases, Cluj Napoca, Romania; ^3^Department of Pharmacology, Toxicology and Clinical Pharmacology, “Iuliu Hațieganu” University of Medicine and Pharmacy, Cluj-Napoca, Romania; ^4^Faculty of Medicine and Pharmacy, University of Oradea, Oradea, Romania; ^5^Clinical Emergency County Hospital, Oradea, Romania; ^6^Department of Dermatology, Faculty of Medicine, “Lucian Blaga” University of Sibiu, Sibiu, Romania; ^7^Clinic of Dermatology, County Emergency Hospital Sibiu, Sibiu, Romania; ^8^Santomar Oncodiagnostic Laboratory, Cluj-Napoca, Romania; ^9^Department of Plastic and Reconstructive Surgery, “Prof Dr. I. Chiricuță” Institute of Oncology, Cluj-Napoca, Romania; ^10^Department of Dermatology, Emergency County Hospital, Cluj-Napoca, Romania

**Keywords:** melanoma, COVID-19 pandemic, prognosis, diagnosis, delay

## Abstract

**Background:**

The COVID-19 pandemic disrupted the healthcare system and negatively affected the diagnosis and management of melanoma worldwide. The purpose of this study is to investigate the long-term effects of the COVID-19 pandemic on the diagnosis and prognosis of melanoma.

**Materials and methods:**

This retrospective cohort study included histopathologically confirmed melanoma cases from March 2019 to February 2023 in Cluj and Bihor counties. Data from the post-COVID-19 period (March 2021 to February 2023) were compared to the pre-COVID-19 period (March 2019 to February 2020) and the COVID-19 period (March 2020 to February 2021). Patient characteristics, monthly diagnostics, histological subtypes, and key histological features were analyzed using statistical tests.

**Results:**

The number of melanoma cases diagnosed annually decreased by 31.37 and 23.75% in the first and second post-pandemic years, respectively, compared to pre-pandemic numbers. Diagnostic rates also decreased by 14.9 and 5.4% in the first and second post-pandemic years, respectively, compared to the pandemic period. Prognostic factors worsened in the post-pandemic period, with higher Breslow index and mitotic rate, and increased ulceration and thick melanomas compared to the pre-pandemic period.

**Conclusion:**

The COVID-19 pandemic had a long-lasting impact on the diagnosis of melanoma in Romania, resulting in advanced stages and unfavorable prognostic factors. Larger global studies are needed to comprehensively understand the pandemic’s long-term effects on the diagnosis of melanoma.

## 1 Introduction

Melanoma, one of the most aggressive types of skin cancers, is a global rising health issue, with a significantly increasing incidence worldwide (1, 2).

The rising incidence might be explained by the frequent exposure to sunlight, increasing number of public screening campaigns and the wide adoption of dermoscopy. Several studies have shown that high sun exposure and indoor ultraviolet (UV) tanning significantly increase the risk of melanoma, while a study conducted in the United States of America (United States) highlighted that the preventive measures are not regularly followed: most of the subjects reported one or more sunburns in the previous year (3–5). Dermoscopy improves the accuracy of diagnosis of melanoma compared to naked eye examination and is widely used in almost all dermatological services. Together with increased patient awareness and frequent public screening campaigns, it allows the detection of melanoma at an earlier stage (6). Even if melanoma is less common than other skin cancers, it remains the most deadly, causing death in all age groups, including adolescents, if diagnosed too late (1, 2).

The survival rate for melanoma varies depending on factors such as the stage of the disease when diagnosed, the Breslow index, the mitotic rate, the presence of tumor ulceration, perineural and lymphatic invasion, the patients’ overall health and the treatment received. For localized melanoma (stage 0-stage I), the 5-year survival rate is around 97%. However, for stage IV melanoma, the five-year survival rate drops to 4% (7, 8). Detecting melanoma in its early stages is crucial for its survival rate as it allows timely intervention and treatment, significantly improving the chances of successful outcome and reducing the risk of severe complications (7, 8).

Threrefore, the early melanoma diagnosis is considered an important task by the healthcare providers worldwide, but the significant rise in melanoma incidence with no increase in mortality might be interpreted as overdiagnosis. Even if the incidence of both invasive and *in situ* melanoma is rising,for *in situ* melanoma it does so exponentially. One possible explanation is that tumors which had previously been diagnosed as “dysplastic nevi” are now labelled as melanoma *in situ*, yet evidence suggests a low probability of progression to invasive melanoma. Another explanation is the rising use of dermoscopy which improves the accuracy of diagnosis (6, 9–11).

The question which arises is whether the overdiagnosis of melanoma has a negative impact on the patient physical and mental health. While most of the patients get a small scar after a wide excision, with almost zero morbidity, the negative emotional and economic effects of overdiagnosis support the efforts to reduce it (12, 13). Nazzaro et al. proposed five dermoscopic predictors of small diameter melanoma to decrease the rate of overdiagnosis and the unnecessary biopsies, but further research on larger datasets is needed to validate the results (14).

Betz-Stablein et al. recommend combining clinical information, total body photography, sequential dermoscopy images and whole slide pathology images associated by artificial intelligence (AI) to increase diagnostic accuracy and decrease the rate of overdiagnosis (9). To date, no healthcare provider should ever take the risk of not excising a lesion just because of the fear of overdiagnosis, as long as the available evidence is insufficient to predict the outcome of the tumor (9, 15).

The most recent and important global pandemic of the last decades, Coronavirus Disease 2019 (COVID-19), caused by the SARS-CoV2 virus, came as a substantial healthcare system challenge. Since the World Health Organization declared the COVID-19 pandemic a global health issue in March 2020, healthcare services had to redirect resources for the management of the COVID-19 patients and most of the countries around the globe imposed harsh restrictions in order to reduce the high infection rates. The restrictions implemented to stop transmission led to drastic reductions in non-urgent medical visits, hindering the management of other conditions (16–18).

The interruption of the healthcare system negatively affected the diagnosis and management of melanoma worldwide. Many studies reported a reduction in the diagnosis of melanoma and an increase in the more advanced stages diagnosed during the COVID-19 pandemic when comparing to the pre-pandemic period (19–23). On the other hand, assessing the cases following the lockdown, they displayed a higher level of progression (24).

Therefore, this study aims to investigate the long-term effects of the COVID-19 pandemic on the diagnosis and prognosis of melanoma in two academic medical centers in Romania (Cluj-Napoca and Oradea).

## 2 Materials and methods

### 2.1 Study design and data sourses

This was an observational, retrospective, cohort study carried out in Cluj and Bihor counties, in the North-Western Region of Romania. We included all the histopathologically confirmed cases of melanoma from 1 March 2019 to 28 February 2023 from all histopathological centers from the two counties (Santomar, Radusan, Emergency County Hospital Cluj, Emergency County Hospital Oradea, Prof. Dr. I. Chiricuta Institute of Oncology Cluj). We compared the results from 1 March 2021 to 28 February 2022 and from 1 March 2022 to 28 February 2023 (the first and second year of the post COVID-19 period) to the pre COVID-19 period (1 March 2019–29 February 2020) and the COVID-19 period (1 March 2020–28 February 2021). We excluded the cases with incomplete data or with a histopathologic diagnosis of melanoma metastasis. This study was approved by the Ethics Committee (No.4249/30.01.2023). Patient characteristics (age and gender), the monthly numbers of melanomas diagnosed, histological subtypes (superficial spreading melanoma (SSM), lentigo maligna melanoma (LMM), nodular melanoma (NM), acral lentiginous melanoma (ALM), and others), and the following histological features (Breslow index, presence of mitosis, ulceration, vascular and neural invasion, and tumor stage [TNM edition]) were collected (25).

### 2.2 Data and statistical analysis

Statistical analysis was performed using the MedCalc® Statistical Software version 20.014 (MedCalc Software Ltd., Ostend, Belgium; https://www.medcalc.org. Numerical variables were expressed as median and 25th–75th percentiles. Categorical data were presented as frequency and percentage. The Mann–Whitney U test was used to determine differences in age, Breslow index, mitotic count and the chi-square test was used for gender, histological subtype, ulceration, vascular/lymphatic invasion, neural invasion, staging. A *p* value <0.05 was considered statistically significant.

## 3 Results

During the study period, 1,284 patients were diagnosed with melanoma (658 females, 626 males). A total of 1,100 patients (341 in the pre-COVID period, 275 in the COVID period and 494 in the post COVID period, 234 between March 2021 and February 2022 and 260 between March 2022 and February 2023) were included in the study. A number of 184 patients were excluded due to missing data. There were no significant differences in patient characteristics in the three cohorts regarding gender. There was a statistically significant difference in the average age for diagnosis: 59 before the pandemic, 63 during the pandemic and 59 after the pandemic.

The total number of melanoma cases diagnosed in the first year of the post COVID19 period decreased by 31.37% and by 23.75% in the second year respectively, when compared to the pre COVID-19 period. The Breslow index and the mitotic rate were statistically significantly higher in the post-COVID-19- first year period (*p* < 0.001) and in the post-COVID19- second year period (*p* = 0.004) when compared to the pre-COVID-19 period. Ulceration was significantly more frequent in the post-COVID-19- first year group (*p* < 0.05). There was also a statistically significantly higher number of thick melanomas in the post-COVID-19 period. (65.0% vs. 49.6% in the first year and 60.0% vs. 49.6% in the second year); *p* < 0.001 and p < 0.05, respectively. The most common histological subtype was SSM in the three periods. A larger number of NMs were diagnosed during the post-COVID-19 period (26.9 and 21.9% vs. 17.6%) (Tables 1, 2).

**Table 1:** Tumor characteristics in the pre-COVID-19 and post-COVID-19- first year period.

Characteristics		PRE COVID (*n* = 341)	POST COVID First year (*n* = 494)	*p*- Value
Median Breslow Index (mm)		1.37 (0.5–3.5)	2.20 (0.7–4.89)	*p* < 0.001
Median Mitotic count (/mm2) (SD)		3 (1–7)	4 (1–10)	0.005
Histological subtype (*n*%)	SSM	258 (75.7%)	152 (65.0%)	0.004
	NM	60 (17.6%)	63 (26.9%)	
	LMM	11 (3.2%)	2 (0.9%%)	
	ALM	8 (2.3%)	10 (4.3%)	
	Other types	4 (1.2%)	7 (3.0%)	
Ulceration	No	229 (67.2%)	132 (56.4%)	0.01
	Yes	112 (32.8%)	102 (43.6%)	
Vascular/ lymphatic invasion (*n*%)	No	324 (95.1%)	214 (91.5%)	0.1
	Yes	17 (4.9%)	20 (8.5%)	
Neural invasion (*n*%)	No	332 (97.4%)	229 (97.9%)	0.9
	Yes	9 (2.6%)	5 (2.1%)	
pT staging (*n*%)	Tis	60 (17.6%)	10 (4.3%)	<0.001
	T1a	86 (25.2%)	57 (24.4%)	
	T1b	26 (7.6%)	15 (6.4%)	
	T2a	34 (10%)	24 (10.3%)	
	T2b	12 (3.5%)	12 (5.1%)	
	T3a	21 (6.2%)	15 (6.4%)	
	T3b	39 (11.4%)	33 (14.1%)	
	T4a	11 (3.2%)	13 (5.6%)	
	T4b	52 (15.2%)	55 (23.5%)	
	Not specified	1 (0.3%)	-	
pT staging group	Thin melanoma (Tis, T1)	172 (50.4%)	82 (35.0%)	<0.001
	Thick melanoma (T2, T3, T4)	169 (49.6%)	152 (65.0%)	
Total		341 (100%)	234 (100%)	

SSM—superficial spreading melanoma, NM—nodular melanoma, LMM—lentigo maligna melanoma, ALM—acral lentiginous melanoma.

**Table 2:** Tumor characteristics in the pre-COVID-19 and post-COVID-19- s year period.

Characteristics		PRE COVID (*n* = 341)	POST COVID Second year (*n* = 494)	*p*- Value
Median Breslow Index (mm)		1.37 (0.5–3.5)	1.40 (0.5–4.2)	0.004
Median Mitotic count (/mm2) (SD)		3 (1–7)	3 (1–8)	0.38
Histological subtype (*n*%)	SSM	258 (75.7%)	186 (71.5%)	0.68
	NM	60 (17.6%)	57 (21.9%)	
	LMM	11 (3.2%)	10 (3.8%)	
	ALM	8 (2.3%)	5 (1.9%)	
	Other types	4 (1.2%)	2 (0.8%)	
Ulceration	No	229 (67.2%)	160 (61.5%)	0.18
	Yes	112 (32.8%)	100 (38.5%)	
Vascular/ lymphatic invasion (*n*%)	No	324 (95.1%)	245 (94.2%)	0.8
	Yes	17 (4.9%)	15 (5.8%)	
Neural invasion (*n*%)	No	332 (97.4%)	255 (98.1%)	0.7
	Yes	9 (2.6%)	5 (1.9%)	
pT staging (*n*%)	Tis	60 (17.3%)	26 (10.0%)	0.012
	T1a	86 (25.2%)	54 (20.8%)	
	T1b	26 (7.6%)	24 (9.2%)	
	T2a	34 (10%)	30 (11.5%)	
	T2b	12 (3.5%)	16 (6.2%)	
	T3a	21 (6.2%)	21 (8.1%)	
	T3b	39 (11.4%)	18 (6.9%)	
	T4a	11 (3.2%)	10 (3.8%)	
	T4b	52 (15.2%)	61 (23.5%)	
	Not specified	1 (0.3%)	-	
pT staging group	Thin melanoma (Tis, T1)	172 (50.4%)	104 (40.0%)	0.014
	Thick melanoma (T2, T3, T4)	169 (49.6%)	156 (60.0%)	
Total		341 (100%)	260 (100%)	

SSM—superficial spreading melanoma, NM—nodular melanoma, LMM—lentigo maligna melanoma, ALM—acral lentiginous melanoma.

The total number of melanoma cases diagnosed in the first year of the post-COVID-19 period decreased by 14.9% and by 5.45% in the second year, respectively, when compared to the COVID-19 period. The Breslow index, the mitotic rate, the percentage of NMs were similar in the post-COVID-19 first year period when compared to the COVID-19 period. The Breslow index and the percentage of NMs were lower in the post-COVID-19 s year when compared to the COVID-19 period, but they do not reach statistical significance. The mitotic rate in the post-COVID-19 s year period was statistically significant lower then in the COVID-19 period (*p* < 0.05). Also, we did not observe a statistically significantly difference in the presence of ulceration, the vascular/ lymphatic invasion, the neural invasion in the post-COVID-19 period (both years) compared to the COVID-19 period. The percentage of thick melanomas was higher in the post-COVID-19 period (both years), but it did not reach statistical significance. However, we observed a statistically significantly difference in melanoma staging between the two periods, more invasive melanomas being diagnosed during the post-COVID-19 period- first year (*p* = 0.007) (Tables 3, 4).

**Table 3:** Tumor characteristics in the COVID-19 and post-COVID-19 first year period.

Characteristics		COVID	POST COVID First year	p- Value
Median Breslow Index (mm) (SD)		2.2 (0.7–5.1)	2.2 (0.7–4.8)	0.2
Median Mitotic count (/mm2) (SD)		4.00 (1–10)	4 (1–10)	0.7
Histological subtype (*n*%)	SSM	188 (68.4%)	152 (65.0%)	0.2
	NM	71 (25.8%)	63 (26.9%)	
	LMM	6 (2.2%)	2 (0.9%)	
	ALM	5 (1.8%)	10 (4.5%)	
	Other types	5 (1.8%)	7 (3.0%)	
Ulceration	No	163 (59.3%)	132 (56.4%)	0.5
	Yes	112 (40.7%)	102 (43.6%)	
Vascular/ lymphatic invasion (*n*%)	No	254 (92.4%)	214 (91.5%)	0.8
	Yes	21 (7.6%)	20 (8.5%)	
Neural invasion (*n*%)	No	269 (97.8%)	229 (97.9%)	1.0
	Yes	6 (2.2%)	5 (2.1%)	
pT staging (*n*%)	Tis	39 (14.2%)	36 (7.3%)	0.007
	T1a	56 (20.4%)	57 (24.4%)	
	T1b	15 (5.5%)	15 (6.4%)	
	T2a	23 (8.4%)	24 (10.3%)	
	T2b	12 (4.4%)	12 (4.4%)	
	T3a	18 (6.5%)	15 (6.4%)	
	T3b	24 (8.7%)	33 (14.1%)	
	T4a	13 (4.7%)	13 (5.6%)	
	T4b	70 (25.5%)	55 (23.5%)	
	Not specified	5 (1.8%)	-	
pT staging group	Thin melanoma (Tis, T1)	115 (41.8%)	82 (35.0%)	0.1
	Thick melanoma (T2, T3, T4)	160 (58.2%)	152 (65.0%)	
	Total	275	234	

SSM—superficial spreading melanoma, NM—nodular melanoma, LMM—lentigo maligna melanoma, ALM—acral lentiginous melanoma.

**Table 4:** Tumor characteristics in the COVID-19 and post-COVID-19 s year period.

Characteristics		COVID	POST COVID Second Year	*p*- Value
Median Breslow Index (mm) (SD)		2.2 (0.7–5.1)	1.40 (0.5–4.2)	0.9
Median Mitotic count (/mm2) (SD)		4.00 (1–10)	3 (1–8)	0.02
Histological subtype (*n*%)	SSM	188 (68.4%)	186 (71.5%)	0.4
	NM	71 (25.8%)	57 (21.9%)	
	LMM	6 (2.2%)	10 (3.8%)	
	ALM	5 (1.8%)	5 (1.9%)	
	Other types	5 (1.8%)	2 (0.8%)	
Ulceration	No	163 (59.3%)	160 (61.5%)	0.6
	Yes	112 (40.7%)	100 (38.5%)	
Vascular/ lymphatic invasion (*n*%)	No	254 (92.4%)	245 (94.2%)	0.4
	Yes	21 (7.6%)	15 (5.8%)	
Neural invasion (*n*%)	No	269 (97.8%)	255 (98.1%)	1.0
	Yes	6 (2.2%)	5 (1.9%)	
pT staging (*n*%)	Tis	39 (14.2%)	26 (10.0%)	0.1
	T1a	56 (20.4%)	54 (20.8%)	
	T1b	15 (5.5%)	24 (9.2%)	
	T2a	23 (8.4%)	30 (11.5%)	
	T2b	12 (4.4%)	16 (6.2%)	
	T3a	18 (6.5%)	21 (8.1%)	
	T3b	24 (8.7%)	18 (6.9%)	
	T4a	13 (4.7%)	10 (3.8%)	
	T4b	70 (25.5%)	61 (23.5%)	
	Not specified	5 (1.8%)	-	
pT staging group	Thin melanoma (Tis, T1)	115 (41.8%)	104 (40.0%)	0.7
	Thick melanoma (T2, T3, T4)	160 (58.2%)	156 (60.0%)	
	Total	275	260	

SSM—superficial spreading melanoma, NM—nodular melanoma, LMM—lentigo maligna melanoma, ALM—acral lentiginous melanoma.

## 4 Discussion

We report the results of the effects of the COVID-19 pandemic on the diagnosis of melanoma in two academic medical centers in Romania 2 years after the pandemic. We found a decrease in the diagnosis of new melanoma of 31.37% in the first year of the post-COVID-19 period and a decrease by 23.75% in the second year, when compared to the pre-COVID-19 period. When compared to the COVID-19 period, we found a 14.9% decrease in the first year and a 5.4% decrease in the second year.

Several studies showed a significant reduction in the diagnosis of new melanomas since the development of the COVID-19 pandemic, which lead to an increase in melanoma diagnosis post-lockdown (19–22). A possible explanation for this finding can be due to the patients’ fear of contacting the disease while exposed to close contact from their face-to-face medical visits, temporary suspension of cancer screening programs and the substitution of face-to-face visits with telemedicine (23). Moreover, operating rooms were used sparingly during the pandemic to limit the transmission of the virus. A study conducted in Italy reported that surgical procedures were longer to allow for proper sanitizing after each patient and therefore, the number of surgeries was lower (20). On the other hand, a study in USA reported that during the pandemic patients experienced longer waiting times from the initial appointment until the diagnostic biopsy when compared to the pre-pandemic patients, but shorter waiting times from the biopsy until the operation (26). During the COVID-19 pandemic, an institution in Canada cancelled all elective surgeries for benign lesions, prioritizing the oncologic surgeries, and found no differences in overall wait time from the consult to the wide local excision surgery in melanoma patients, when compared to the pre-pandemic period (27). In our region we were able to continue dermo-oncologic surgery throughout the entire lockdown and pandemic period, but similar to the study by Gauldi et al. (20), surgical procedures were longer and the number of surgeries was reduced. Furthermore, we noted that many patients spontaneously cancelled their appointments for fear of getting infected.

Interestingly, our study shows that fewer cases were diagnosed each year after the COVID-19 pandemic. Even if a trend towards normality was observed between the first and the second year of the post COVID-19 period (a 10% increase of newly diagnosed melanoma cases), the diagnostic rates have not yet fully resumed. However, other studies support our findings. The study conducted by Jeremic et al. reported lower melanoma diagnosis rates in 2021 and 2022 when compared to the pre-pandemic years (28). Another Serbian study found 65 melanoma patients registered in 2018, 31 in 2019, 16 in 2020, 30 in 2021, 47 in 2022 and 10 in the first three months of 2023 (29). In Romania, a study by Aabed et al. reported a 17% reduction in melanoma cases 24 months after the outbreak of the COVID19 pandemic when compared to the 24 months before the pandemic, which means that even in the pandemic recovery phase, patients are still reluctant to ask for medical care (30). Also, the decrease in the number of cases after the pandemic in our study might be explained by the fact that during the pandemic there were much fewer skin cancer prevention, screening and awareness campaigns and by the tendency of patients to ask for medical care only in severe situations.

The delay in the diagnosis of melanoma resulted in worse prognostic factors. Compared to the pre-COVID period, the Breslow index was significantly higher during the post-COVID-19 period. In the first year after the COVID-19 pandemic, the mitotic rate was significantly higher and the presence of ulceration was more frequent, which subsequently led to an increase in the number of thick melanomas. Fewer patients presented with *in situ* disease post-COVID-19, 4.3% in the first year and 10.0% in the second year vs. 17.3% pre-COVID-19 and a higher proportion presented with thick melanomas 65.0% in the first year and 60.0% in the second year vs. 49.6%. Compared to the COVID-19 period, the Breslow index did not reach statistical significance in the post COVID-19 period (both years) and there was no statistically significant difference in the presence of ulceration, vascular/ lymphatic invasion, and neural invasion between the three periods. NMs, the histological subtype with the worst survival rates, increased in the post-COVID-19- first year period when compared to the pre-COVID-19 period and remained constant when compared to the post-COVID-19- s year and COVID-19 period (31).

The shift to more invasive melanomas was documented by other studies (32–34). Gualdi et al. evaluated melanomas excised within two months after the end of the lockdown and found poorer prognostic factors of the disease such as: a higher Breslow index, a higher number of mitoses and a higher number of ulcerated melanomas (20). Another study carried out in Spain compared the melanoma diagnosed one year before the lockdown and one year after the lockdown and found an increase in Breslow thickness, as well as an increase in the number of mitoses and in the percentage of ulcerated melanomas (35). A systematic review carried out by Toma AO et al. analyzing the epidemiology of melanoma 2 years after the COVID-19 pandemic found that the percentage of thick melanomas was higher during 2020–2021 then before the pandemic (36.25% *vs* 25.88%). They also found a decrease in the proportion of *in situ* melanoma diagnoses, reported by most of the studies included in the review (36).

On the other hand, an Austrian study reported statistically significant differences in the presence of ulceration in the year after the lockdown, but there were no differences in the Breslow index (19). A nationwide study developed in the Netherlands revealed only a minor shift towards more advanced melanoma stages during the first lockdown and no impact afterwards (37). Klepfisch et al. found no clinical or histopathological differences in the number of melanomas diagnosed after the lockdown compared to the pre-lockdown period and reported a minimal impact of COVID-19 lockdown on the diagnosis of melanoma, however, other studies reported the contrary (38). One possible explanation for the difference between data reported in different countries could be the different magnitude of the pandemic throughout the world (21, 24, 39).

Unfortunately, the effects of the COVID-19 pandemic affect not only those who presented in the months following the lockdown. As was feared, a trend towards a higher cancer stage at presentation still remains for patients in the post-COVID-19 period.

Melanoma represents one of the most aggressive forms of the routinely monitored cancers. More precisely, the doubling time for melanoma is 94 days compared to 936 days for colorectal adenocarcinoma (40, 41). Thus, early diagnosis is crucial for melanoma-associated survival as the risk of metastases increases with a higher Breslow index, mitotic rate and presence of ulceration (42). The COVID-19 pandemic postponed most cancer diagnosis in the corresponding period and therefore postponed treatment. Tejera et al. published a rate of growth model and calculated a 2% loss in 5-year survival for melanoma patients diagnosed with a delay of up to 3 months (43). Another study found that melanoma patients who are diagnosed with a 3-month delay experience a reduction in five-year survival rates of 1.9%, and ten-year survival of 2.4%. Moreover, it has been estimated that melanoma patients diagnosed after March 2020 have a 7% reduction in their survival rates (44). Our study found a higher Breslow index and a greater percent of thick melanomas in the post-COVID period. If melanomas were indeed growing as fast as previously reported, this effect would have been observed only in the period immediately after lockdown. This might suggest that melanomas may grow slower than previously assumed or only the slow-growing tumors were delayed in terms of being diagnosed (37).

Furthermore, patients diagnosed before and after the pandemic were younger than those diagnosed during the pandemic (59 vs. 63 years old), suggesting that elderly patients did not underestimate the severity of their disease. On the other hand, Hurley et al. reported that the mean age was statistically significantly higher pre-COVID-19 compared to the COVID-19 period (68 vs. 63 years), and Martinez- Lopez et al. described a median age of 77 years in the pre-pandemic period compared to 53 years during 2020 and 2021, which might mean that the elderly are more concerned about contacting the COVID-19 (35, 45). A study conducted by Asai et al. reported no difference in the median age of the cohort, both before and during the pandemic (46).

Regarding cancer in general, a study carried out in Catalonia saw a substantial reduction in cancers over two pandemic years (until January 2022), with an estimated 13,000 undetected cancers, compared with the preceding year. Although a trend towards normality was observed in 2021, the diagnostic rates have not yet fully recovered. Also, they found that the pandemic affected women less than men. When it comes to our study, there was no statistically significant difference between the two genders and other studies support our findings (47–49).

Most of the countries worldwide adopted harsh measures in order to prevent the spreading of the COVID-19 and meetings and crowded places had to be avoided until complete virus extinction. Therefore, melanoma screening campaigns, where usually a lot of people took part in, were cancelled. Even if the restrictions were lifted and the screening programs restarted, these delays in screening created a “bottleneck effect” and large numbers of melanomas are still undiagnosed (48). Dermatology specialists, oncology specialists and primary care providers should encourage patients to go back to the doctor and resume their screening programs (49). Moreover, healthcare providers should work together in order to promote and organize more screening campaigns. These may include public awareness campaigns, promoting whole-body self-examination, patient education about sun protection, media campaigns and in person screening programs (50).

To our knowledge, this is the first study that reports the effects of the COVID-19 pandemic on the diagnosis of melanoma 2 years after the pandemic. The strengths of this study lie in the collection of homogeneous data and the long time frames. The limitations of our study consist of it being a retrospective study, the data was not collected from patients seen during the three periods. In addition, it is a double center-study and the sample is limited.

## 5 Conclusion

Our study suggests that the COVID-19 pandemic negatively affected the diagnosis of melanoma 2 years after the COVID-19 pandemic in Romania, leading to the detection of the tumor at more advanced stages and worse prognostic factors, which require more diagnostic procedures and more aggressive treatments, resulting in greater healthcare costs and worse survival rates for these patients. Even if the normal clinical activity was restored after the COVID-19 lockdown, our study shows that the diagnostic rates of melanoma have not yet fully recovered. Health care professionals should work together in order to encourage patients to resume their normal medical visits. We believe that larger worldwide studies are needed in order to better define the long-term effects of the COVID-19 pandemic on the diagnosis of melanoma.

## Data availability statement

The raw data supporting the conclusions of this article will be made available by the authors, without undue reservation.

## Ethics statement

The studies involving humans were approved by Ethics Committee of the Emergency County Hospital Cluj. The studies were conducted in accordance with the local legislation and institutional requirements. Written informed consent for participation was not required from the participants or the participants' legal guardians/next of kin in accordance with the national legislation and institutional requirements.

## Author contributions

AA: Conceptualization, Data curation, Investigation, Methodology, Writing – original draft. ȘV: Methodology, Software, Writing – review & editing. SF: Data curation, Formal analysis, Writing – original draft. GI: Data curation, Formal analysis, Writing – original draft. NB: Data curation, Formal analysis, Writing – original draft. MM: Data curation, Formal analysis, Writing – original draft. SȘ: Data curation, Formal analysis, Investigation, Writing – original draft. OB: Data curation, Formal analysis, Writing – original draft. CS: Data curation, Formal analysis, Writing – original draft. LU: Conceptualization, Methodology, Supervision, Validation, Visualization, Writing – review & editing.
